# Mapping indicators of tobacco and related product use: Unveiling challenges and variations in the Eurobarometer surveys over three decades

**DOI:** 10.18332/tid/202651

**Published:** 2025-04-16

**Authors:** Ayaka Teshima, Cristina Martínez, Filippos T. Filippidis, Anthony A. Laverty, Constantine I. Vardavas, Ariadna Feliu, Armando Peruga, Esteve Fernandez

**Affiliations:** 1 Tobacco Control Unit, WHO Collaborating Centre for Tobacco Control, Catalan Institute of Oncology (ICO), L’Hospitalet de Llobregat, Barcelona, Spain; 2 Tobacco Control Research Group, Bellvitge Biomedical Research Institute-IDIBELL, L'Hospitalet de Llobregat, Barcelona, Spain; 3 School of Medicine and Health Sciences, University of Barcelona, Barcelona, Spain; 4 School of Nursing, University of Barcelona, Barcelona, Spain; 5 Centro de Investigación Biomédica en Red de Enfermedades Respiratorias (CIBERES), Madrid, Spain; 6 Department of Primary Care and Public Health, School of Public Health, Imperial College London, London, United Kingdom; 7 Department of Hygiene, Epidemiology and Medical Statistics, Medical School, National and Kapodistrian University of Athens, Athens, Greece; 8 Environment and Lifestyle Epidemiology Branch, International Agency for Research on Cancer (IARC/WHO), Lyon, France; 9 Centro de Epidemiología y Políticas de Salud, Facultad de Medicina, Clínica Alemana Universidad del Desarrollo, Santiago, Chile; 10 Catalan Health Department, Government of Catalonia, Barcelona, Spain

**Keywords:** Eurobarometer, smoking indicators, tobacco and related products

## Abstract

**INTRODUCTION:**

The European Commission’s Special Eurobarometer surveys on tobacco are widely used as comparable and representative data across the European Union for monitoring consumption patterns. Despite their broad use, certain challenges persist, including inconsistencies in survey timelines and variations in the collected information across waves. This study aims to identify available tobacco and related product indicators, and assess their temporal comparability, to support researchers to better understand the potential uses of these data and their alignment with other sources.

**METHODS:**

We explored questionnaires and reports in these surveys on tobacco from the Eurobarometer official website since its inception (1992, 2002, 2005, 2006, 2008, 2009, 2012, 2014, 2017, 2020 and 2023 waves). We extracted and compared questions and responses on use of tobacco and related products, as well as on sociodemographic variables. Finally, we compared all indicators, including frequency and wording, and further mapped the consistency of the indicators and type of product used across the different waves.

**RESULTS:**

Current, daily, former and never use of conventional cigarettes has been consistently assessed across all waves, enabling temporal comparisons. From 2009, the surveys have expanded to include e-cigarette use; since 2012, the surveys include various combusted products such as cigars, pipes, cigarillos, and waterpipes, and from 2020, heated tobacco products. By contrast, detailed data on product-specific use intensity and initiation remain limited. While indicators for secondhand smoke exposure and smoking cessation were present in multiple waves, their comparability is hindered by variations in question-wording and responses.

**CONCLUSIONS:**

Some challenges exist in using Eurobarometer surveys for temporal estimation of tobacco-related burden. Nonetheless, these surveys remain a valuable and unique tool for monitoring tobacco and related product use across the European Union. To further enhance their utility, periodic re-evaluation by tobacco control experts is recommended to ensure that the surveys maintain comparability with past data while capturing effectively emerging products and trends.

## INTRODUCTION

Accurate and rigorous surveillance is crucial for monitoring and tracking tobacco and related product consumption patterns to implement effective tobacco control measures. Monitoring the use of tobacco and related products is a vital component of MPOWER (Monitor, Protect, Offer, Warn, Enforce, Raise), a technical package of selected demand reduction measures based on the World Health Organization (WHO) Framework Convention on Tobacco Control (FCTC). Monitoring is critical for evaluating the success of MPOWER and the WHO FCTC^1^.

The European Union (EU) has addressed the tobacco epidemic for decades through measures such as ratifying the WHO FCTC and enacting directives such as the Tobacco Advertising Directive (2003/33/EC), Tobacco Tax Directive (2010/12/EU), Audio-visual Media Services Directive (2010/13/EU) and the Tobacco Products Directive (2014/40/EU)^1,2^. In 2021, the Europe’s Beating Cancer Plan has set a goal for a ‘Tobacco-Free Generation’, aiming for <5% of the population to use tobacco by 2040^3^. Despite these efforts, the EU, with a population of more than 450 million, still has a high prevalence of current tobacco smoking (21% in women and 28% in men, in 2023)^4^. The 2019 Global Burden of Disease Study estimated that over 923000 annual deaths in the EU from smoking and secondhand smoke (SHS) exposure, representing approximately 17% of all deaths^5^.

While tobacco use prevalence is the primary indicator of current trends^6^, incorporating a range of intermediate measures offers a deeper understanding of changing behaviors toward tobacco and related products, as well as a more comprehensive assessment of policy impacts^7-9^. These include consumption patterns, patterns and type of product used, smoking intensity, former smoking behavior, nicotine dependence, cessation, and initiation of smoking^9-11^. Such measures are crucial for predicting health risks, evaluating policy success, and understanding the impact on healthcare costs, revenues from tobacco taxes, industry financing and priorities, and public health resources^12,13^.

Population-based robust data are essential for tracking long-term trends and enabling regional and global comparisons. Leading surveillance tools for cross-national comparisons of tobacco and related product use include the WHO Global Adults Tobacco Survey (GATS)^14^, the European Health Interview Survey (EHIS)^15^, and the International Tobacco Control Policy Evaluation (ITC) Project^16^. Within the EU, national surveys lack comparability due to differing methodologies and frequencies^17^.

Since 1973, the European Commission (EC) Eurobarometer surveys have collected data across EU Member States (MS) on public opinion and attitudes toward various issues, including European institutions, policies, and the integration process^18^. The Standard Eurobarometer comprises two yearly surveys (each autumn and spring), whereas the Special Eurobarometer surveys are in-depth thematic studies relevant to the activities of European institutions. The Special Eurobarometer on tobacco use, first launched in 1992^18^, provides data on the use of tobacco and related products. Despite a relatively small sample size per MS (typically around 1000 participants), the use of standardized methods ensures that these data remain comparable and representative across the EU, providing valuable insights into tobacco use and tobacco control measures^4^.

Given the evolving landscape of tobacco control and the emergence of new tobacco and related products, using diverse indicators and accounting for different product types to assess the tobacco epidemic is important^19^. This approach could help to accurately estimate and compare the population-level burden of smoking over time. Thus, periodic reviews of these surveys on tobacco are needed. This study aims to identify and classify available indicators of tobacco and related products across Eurobarometer waves and assess their comparability over time.

## METHODS

### Study design, setting and participants

Two reviewers (AT and CM) searched the Eurobarometer website^18^ using the keyword ‘tobacco’, which yielded 30 records. We excluded ten records that were not directly related to the use of tobacco or related products. Additionally, records about public perception of illicit tobacco trade/cross-border shopping (three records) and, a qualitative survey about tobacco packaging health warnings (one record), and another on youth attitudes on drugs (five records) were excluded since the information was not directly related to our objective. Ultimately, we selected 11 records for the study.

We applied a scoping review approach^20^ using data sources from waves 38.0 (1992), 58.2 (2002), 64.1 (2005), 66.2 (2006), Flash 253 (2008), 72.3 (2009), 77.1 (2012), 82.4 (2014), 87.1 (2017), 93.2 (2020), and 99.3 (2023) of these surveys’ questionnaires and reports. The 1992 wave was a regular Eurobarometer, while all subsequent waves, except for the 2008 Flash survey, were Special Eurobarometer. The Flash Eurobarometer conducts rapid surveys on specific topics, using phone interviews, to quickly gather opinions on urgent or time-sensitive matters. These surveys, conducted by the EC, have a population-based cross-sectional design with representative samples of individuals aged ≥15 years. Tables 1 and 2 show the technical characteristics of these Eurobarometer surveys, including the wave (year conducted), fieldwork period, numbers of countries included, total sample size, and country list. As the questionnaire on sociodemographic information for the 2023 wave has not yet been released, the analysis for sociodemographic information was limited to surveys conducted between 1992 and 2020.

**Table 1:** Key methodological details of Eurobarometer surveys on tobacco use (11 waves: 1992–2023)

*Wave (year)*	*Official reference of Eurobarometer (EB)*	*Field work period*	*Number of countries (EU MS)*	*Sample size*
1992	EB 38.0	21 Sep – 9 Oct	12 (12)	12800
2002	Special EB 58.2	28 Oct – 8 Dec	15 (15)	26000
2005	Special EB 64.1- 64.3	2 Sep – 7 Dec	30 (25)	24643
2006	Special EB 66.2	6 Oct – 8 Nov	29 (25)	28584
2008	Flash EB 253	13 – 17 Dec	27 (27)	26582
2009	Special EB 72.3	2 – 19 Oct	31 (27)	30292
2012	Special EB 77.1	25 Feb – 11 Mar	27 (27)	26751
2014	Special EB 82.4	29 Nov – 9 Dec	28 (28)	27801
2017	Special EB 87.1	18 – 27 Mar	28 (28)	27901
2020	Special EB 93.2	3 Aug – 15 Sep	28 (28)	28300
2023	Special EB 99.3	10 May – 5 Jun	27 (27)	26358

MS: EU Member States.

**Table 2:** Country coverage of Eurobarometer tobacco surveys (11 waves: 1992–2023)

*No.*	*EB wave (year)*	*1992*	*2002*	*2005*	*2006*	*2008*	*2009*	*2012*	*2014*	*2017*	*2020*	*2023*
	Countries	12	15	30	29	27	31	27	28	28	28	27
	EU MS	12	15	25	25	27	27	27	28	28	28	27
1	Belgium	■	■	■	■	■	■	■	■	■	■	■
2	Denmark	■	■	■	■	■	■	■	■	■	■	■
3	Germany	■	■	■	■	■	■	■	■	■	■	■
4	Greece	■	■	■	■	■	■	■	■	■	■	■
5	Spain	■	■	■	■	■	■	■	■	■	■	■
6	France	■	■	■	■	■	■	■	■	■	■	■
7	Ireland	■	■	■	■	■	■	■	■	■	■	■
8	Italy	■	■	■	■	■	■	■	■	■	■	■
9	Luxembourg	■	■	■	■	■	■	■	■	■	■	■
10	Netherlands	■	■	■	■	■	■	■	■	■	■	■
11	Portugal	■	■	■	■	■	■	■	■	■	■	■
12	United Kingdom	■	■	■	■	■	■	■	■	■	■	-
13	Austria		■	■	■	■	■	■	■	■	■	■
14	Finland			■	■	■	■	■	■	■	■	■
15	Sweden			■	■	■	■	■	■	■	■	■
16	Czechia			■	■	■	■	■	■	■	■	■
17	Estonia			■	■	■	■	■	■	■	■	■
18	Rep. of Cyprus			■	■	■	■	■	■	■	■	■
19	Latvia			■	■	■	■	■	■	■	■	■
20	Lithuania			■	■	■	■	■	■	■	■	■
21	Hungary			■	■	■	■	■	■	■	■	■
22	Malta			■	■	■	■	■	■	■	■	■
23	Poland			■	■	■	■	■	■	■	■	■
24	Slovenia			■	■	■	■	■	■	■	■	■
25	Slovakia			■	■	■	■	■	■	■	■	■
26	Bulgaria			◇	◇	■	■	■	■	■	■	■
27	Romania			◇	◇	■	■	■	■	■	■	■
28	Croatia			◇	◇	-	◇	-	■	■	■	■
29	Turkish Cypriot Comm.			◇	◇	-	◇	-				
30	Turkey			◇	-	-	◇	-				
31	Former Yugoslav Rep. of Macedonia						◇	-				

■ Countries that were already members of the EU at the time of the survey. ◇ Countries that were not part of the EU at the time of the survey but were included in the survey. MS: EU Member States.

### Data extraction and item scoping analysis

Two reviewers (AT and CM) manually extracted all information on tobacco and related product indicators and sociodemographic variables from each wave of the Eurobarometer surveys. If discrepancies arose, the reviewers discussed them until consensus. If no agreement was achieved, a third reviewer (EF) was consulted to resolve the differences.

Direct tobacco and related product indicators were categorized into four sections: 1) Patterns of use, 2) Secondary exposure, 3) Smoking cessation, and 4) Knowledge, attitudes, and perceptions (KAP). Tobacco and related products were divided into four main categories: combustible tobacco, oral/nasal tobacco, non-combustible tobacco, and non-tobacco nicotine products. If a questionnaire item asked about a specific tobacco or related product, especially regarding patterns of use, we categorized the data for that product individually. However, if a questionnaire item asked about a group of tobacco or related products, we categorized the data for that entire group. For example, some waves had a questionnaire item specifically about cigar use, while others grouped cigars and pipe tobacco together in a single questionnaire item.

To analyze how comparable the data were across different waves, we also identified derived proportions and their numerators and denominators that can be calculated from the available direct questionnaire information. Direct data are derived directly from the responses to a single survey question (e.g. prevalence of manufactured cigarettes smoking). In contrast, derived indicators are computed by combining information from two or more survey questions (e.g. prevalence of smoking as the number of people who smoke the different combustible products divided by the total population). Finally, we compared all tobacco and related product information and sociodemographic characteristics across the waves, including frequency and wording, and further mapped their consistency and evolution over time.

### Ethical approval

No ethical approval from an ethics committee was required for this study since the data sources, including questionnaires are publicly available through the EC^18^. The study does not use these Eurobarometer datasets with the individual microdata.

## RESULTS

Table 3 compares the direct data on patterns of use by various tobacco and related products, along with their possible definitions between 1992 and 2023. Supplementary file Table 1 provides the wording of specific questions for each tobacco and related product item.

**Table 3:**
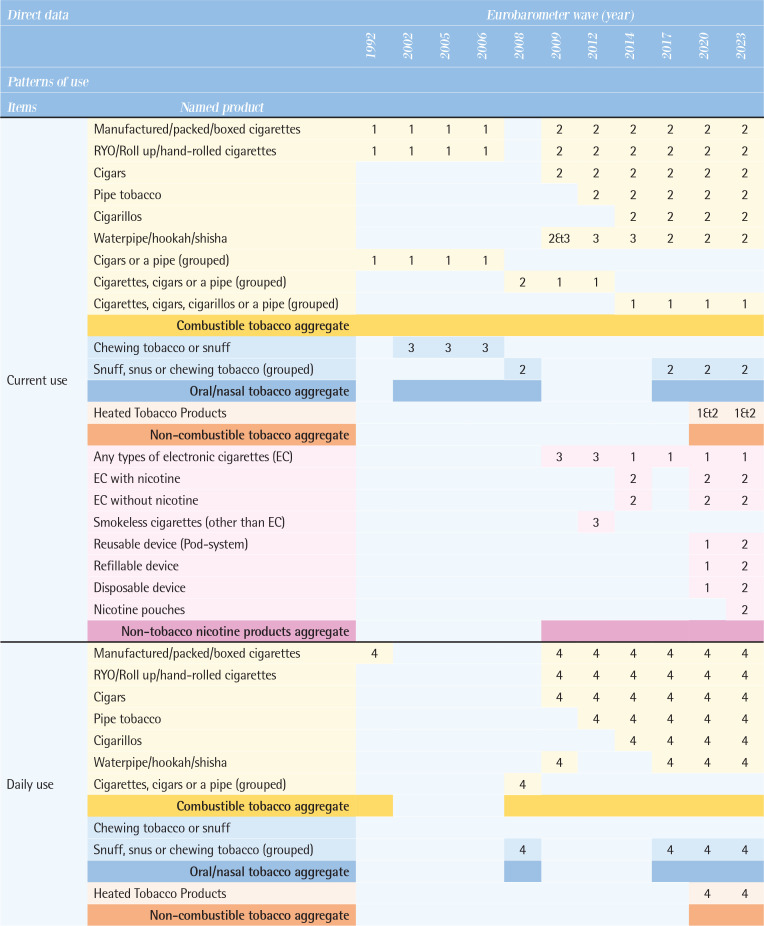
Comparison of tobacco and related products use patterns by types of products across the Eurobarometer waves (11 waves: 1992–2023)

**Figure:**
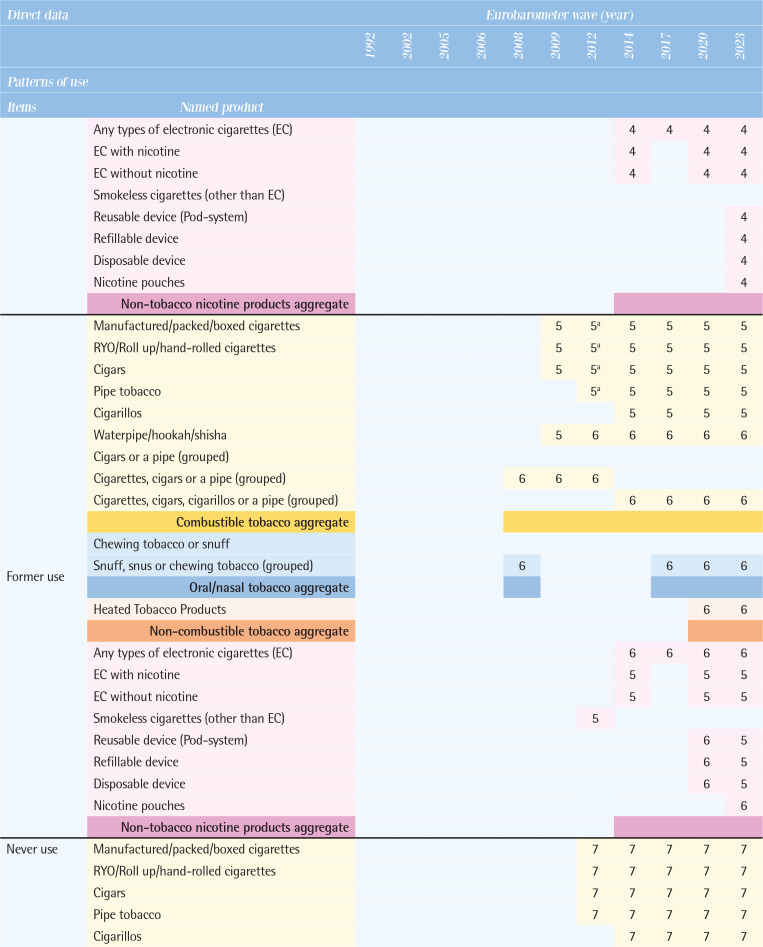


**Figure:**
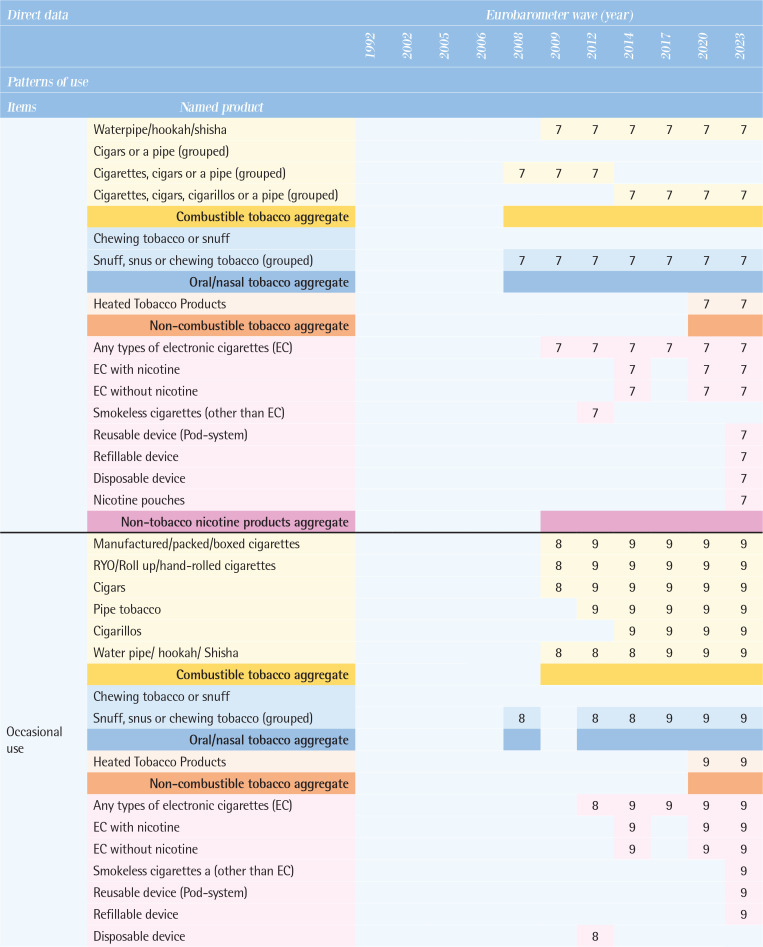


**Figure:**
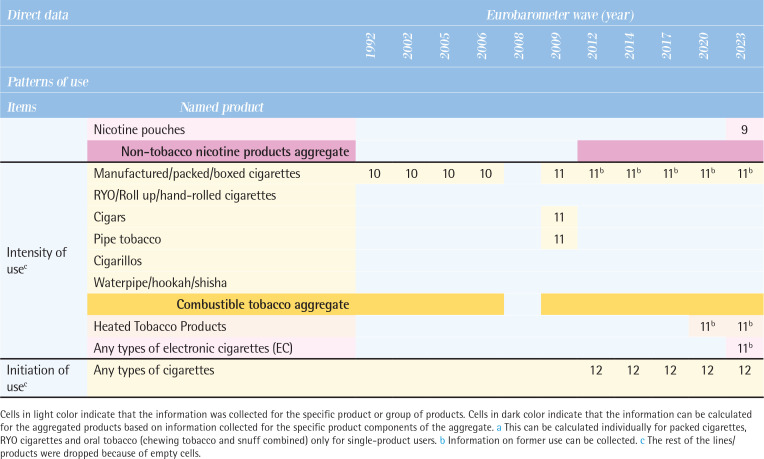

Interpretation of numbers in Table 3: classification of indicators by Eurobarometer surveys response types:

1. Smoke at present time or currently smoke or currently use.
2. Smoke or use product every day.
3. Smoke or use regularly.
4. Smoke every day/use product every day or take product every day.
5. Did use product every day or occasionally or weekly or monthly or <monthly.
6. Used to smoke but have stopped/used to take it regularly but stopped.
7. Never used product/never smoked.
8. Smoke occasionally/smoke or use occasionally.
9. Use product <monthly.
10. <5/5–9/10–14/15–19/20–24/ 25–29/ 30–34/ 35–39/≥40 cigarettes in a day.
11. Continuous number/units of cigarettes to smoke or use products each day.
12. Age at starting smoking on a regular basis.

### Types of tobacco and related products used

All tobacco and related products in these surveys were categorized into four groups. First, combustible tobacco products included manufactured cigarettes, roll-your-own (RYO) cigarettes, cigars, cigarillos, pipe tobacco, and waterpipe/hookah. Some questions grouped products together, such as ‘cigars or a pipe’ ‘cigarettes, cigars or a pipe’, and ‘cigarettes, cigars, cigarillos or a pipe’. Combustible tobacco products were generally included in all waves, but the breakdown by specific product was not always available. Second, oral/nasal tobacco products, which were asked about as ‘chewing tobacco or snuff’ or ‘snuff, snus, or chewing tobacco’, showed the most variation across the waves. Third, the non-combustible Heated Tobacco Products (HTPs) have been included in the questionnaires since the 2020 wave. Fourth, non-tobacco nicotine products, encompassed any types of e-cigarettes and similar products. Use of any types of e-cigarettes has been asked since the 2009 wave. Newer products, such as reusable devices, refillable devices, and disposable devices, are included since the 2020 wave, and nicotine pouches since the 2023 wave.

### Patterns of use

According to the questions and responses option, possible definition on patterns of use were identified for each item (see footnote of Table 3). The questions actually used for each item are shown in Supplementary file Table 1.

*Current use*

The wording of the questions on tobacco use has changed slightly and varied from the 1992 to the 2008 waves, at times inquiring about smoking status at the time of the survey and at other times asking whether participants smoke regularly, daily, or occasionally. From the 2009 wave onwards, the answers were consistent, with three categories: ‘You currently smoke’, ‘You used to smoke, but you have stopped’, and ‘You have never smoked’. There is a significant variation by product. Data on the current use of manufactured cigarettes and RYO cigarettes have been collected in all waves except 2008; however, data on the current use of cigars have been collected since the 2009 wave, pipe tobacco since the 2012 wave, and cigarillos since the 2014 wave. Data on current use of oral tobacco, continuously collected from the 2002 wave to the 2008 wave, were absent from the 2009 wave to the 2014 wave, but resumed in the 2017 wave. Data on e-cigarettes have been assessed consistently since the 2009 wave, but detailed product information (e.g. nicotine content) has varied across different survey waves. Data on current use of HTPs and detailed e-cigarette types (reusable devices, refillable devices, and disposable devices) have been included since 2020, and nicotine pouch use was newly assessed in the 2023 wave.

*Former use*

There was little variation in the wording of questions. The question did not specify a period of abstinence from smoking, and responses such as ‘You used to smoke but you have stopped’ or ‘You used to use it, but you have stopped’ were used. Until the 2008 wave, products were not specified and were asked about under the category of combustible tobacco or as ‘cigarettes, cigars, or a pipe (grouped)’. Since the 2009 wave, data on manufactured cigarettes, RYO, and cigars have been consistently assessed, with cigarillos assessed from the 2014 wave onwards. Data on former use of pipe tobacco and waterpipes has been collected inconsistently. For e-cigarettes, former use has been assessed since the 2014 wave, and HTPs since the 2020 wave.

*Never use*

Comparisons within the combustible tobacco category of never use have been consistent across all waves. Since the 2012 wave, data on never use has been assessed by each product, including manufactured cigarettes, RYO, cigars, cigarillos, pipe tobacco, and waterpipe, allowing for product-specific evaluation. Data on never use of oral tobacco has been continuously assessed since the 2008 wave as snuff, snus or chewing tobacco (grouped), for e-cigarettes since the 2012 wave, and HTPs since the 2020 wave.

*Frequency of use*

Daily use

There was variation in the wording of questions (Supplementary file Table 1) and products specified: manufactured cigarettes (the 1992 wave), unspecified cigarettes products (the 2002–2006 waves), and ‘cigarettes, cigars, or a pipe (grouped)’ (the 2008 wave). Since the 2009 wave, data on daily use of manufactured cigarettes, RYO, and cigars have been consistently collected. For e-cigarettes, daily use has been assessed since the 2014 wave, and HTPs since the 2020 wave.

Occasional use

Product-specific distinctions were not possible between the 2002 and 2008 waves; the data can be assessed under combustible tobacco products. However, since the 2009 wave, data on frequency of use have been consistently collected for manufactured cigarettes, RYO, cigars, and waterpipe, for pipe tobacco and e-cigarettes since the 2012 wave, cigarillos since the 2014 wave, and HTPs since the 2020 wave.

*Intensity of use*

Data on current number of cigarettes smoked per day have been assessed in all waves except for the 2008 waves, while data on the past number of cigarettes per day have been assessed from the 2012 wave onwards, but not from earlier waves. In the 2009 wave, number of cigarettes smoked per day was assessed separately for manufactured cigarettes, cigars, and pipe tobacco, although types of combustible tobacco products were not distinguished in the rest of the waves. Between the 1992 and 2006 waves, for the question: ‘Do you smoke every day? If so, how many cigarettes a day do you smoke?’, the response was incorporated as a categorical variable in pre-determined group answers rather than as continuous variable. After the 2009 wave, the questionnaire was changed to fill in a continuous variable. From the 2020 wave onwards, consumption related to HTPs was asked, and in the 2023 wave, a question on the number of times e-cigarettes used was included.

*Initiation of use*

Data on age of smoking initiation have been consistently assessed in all waves since 2012, but not in earlier waves. Throughout the surveys, respondents were consistently asked: ‘How old were you when you started smoking on a regular basis, i.e. at least once a week?’. Since no distinction was made between products, the reported initiation age of regular smoking can be applied only to a broad category of combustible tobacco products and does not capture the age of first experimentation with smoking. Table 4 illustrates direct data related to tobacco and related products and sociodemographic indicators.

**Table 4:**
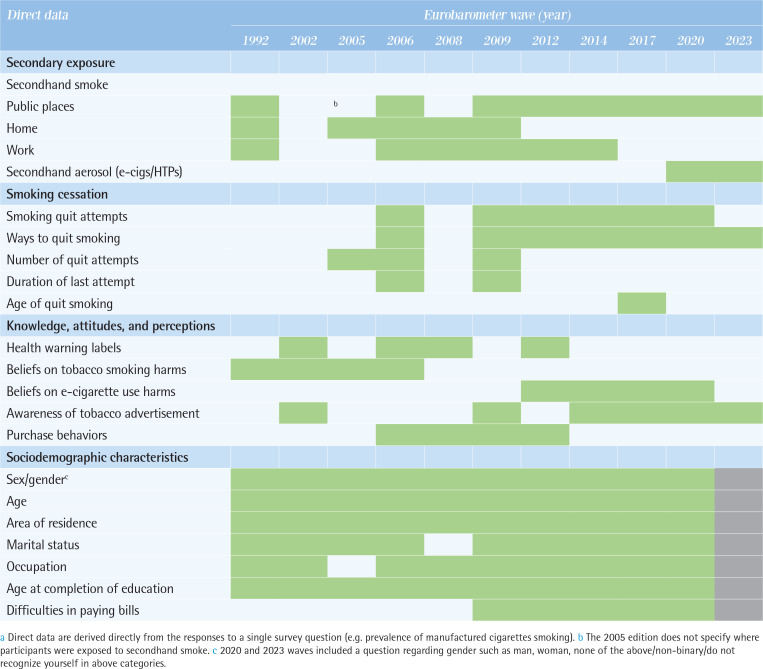
Direct data^a^ related to tobacco and related products and sociodemographic characteristics across the Eurobarometer waves (11 waves: 1992–2023)

### Involuntary or passive exposure

Data on secondhand smoke (SHS) exposure greatly varied in terms of locations where SHS exposure occurred and the wording of questions across the waves (Table 4, and Supplementary file Table 1). SHS-related information did not distinguish between products and was assessed under the category of combustible tobacco. Data on SHS exposure at home was only assessed in the 1992 and 2005–2009 waves, and for SHS exposure at the workplace, it was assessed in the 1992 and 2006–2014 waves. Data on SHS exposure in public places has been assessed since the 1992 and 2006 waves, and consistently since 2009. While the locations where SHS exposure was assessed were mainly public places such as bars and restaurants, other outdoor areas of restaurants, concerts, and spaces intended for children and youth were included from the 2020 wave onwards.

In the 1992 wave, respondents were asked if there were smokers in the public places they visited; in the 2005 wave, they were asked the duration of exposure to smoke (>5, 1–5, <1 hour a day); and in 2006 wave, in addition to the duration, they were asked about the frequency of SHS exposure (very often or sometimes). Until the 2006 wave, there were no specific limitations on the period of SHS exposure. Since the 2009 wave, data on SHS exposure have been assessed based on whether respondents had seen smokers indoors (e.g. bars and restaurants) within the past six months.

Data on secondhand aerosol (SHA) from e-cigarettes and HTPs were collected in the 2020 and 2023 waves. Questions about SHA included the following locations: indoor public spaces (e.g. restaurants, bars, shopping malls, airports, concert halls), outdoor spaces intended for use by children or adolescents (e.g. nursery and school courtyards, playgrounds), public spaces (e.g. parks, beaches, entrances to public buildings), and open-air public transportation stations (e.g. bus, tram, or train stations).

### Smoking cessation

Data related to smoking cessation, such as smoking quit attempts, methods used to quit smoking, number of quit attempts in the last 12 months, duration of last quit attempt, and age at quitting, were extracted, but these indicators were not consistently measured and were diverse across the waves (Table 4, and Supplementary file Table 1). None of these indicators distinguished between products and were assessed under the category of combustible tobacco. The number of quit attempts in the last 12 months (the 2005, 2006, and 2009 waves), the duration of the last quit attempt (the 2006 and 2009 waves), and the age at quitting (the 2017 wave) were assessed occasionally. Data on smoking quit attempts were continuously collected from the 2005 and 2006 waves, and between the 2009 and 2020 waves, but were omitted in the 2023 wave. The wording of the questions also varied; in the 2005, 2006 and 2009 waves, the number of quit attempts was categorized as: 1–5, 6–10, and >10 times in the last 12 months). Since the 2012 wave, the definition changed to whether an attempt to quit was made within the past 12 months or more than a year ago. In the 2020 wave, data on quit attempts using e-cigarettes and HTPs were assessed.

### Knowledge, attitudes, and perceptions

Information on health warning labels was assessed from questions on what the respondents thought of the effectiveness of health messages on tobacco packs (see the actual questions in Supplementary file Table 1). Beliefs about tobacco smoking harms, beliefs about e-cigarette smoking harms, awareness of tobacco advertisements, and purchase behaviors were also abstracted in the section. All items were assessed occasionally (Table 4).

### Sociodemographic variables

Supplementary file Table 2 illustrates changes in the wording of questions related to sociodemographic and socio-economic status information. While the wording of marital status varied widely, consistent wording was maintained for sex, age, area of residence, age at completion of formal education, and occupation, in most cases over time. Data on financial difficulties have been assessed in all waves since the 2009 wave.

### Derived indicators

Table 5 illustrates derived indicators related to tobacco and related products.

**Table 5:**
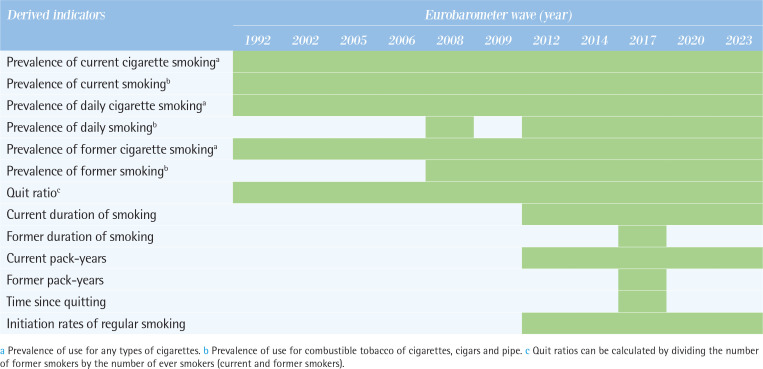
Derived indicators related to tobacco and related products across the Eurobarometer waves (11 waves: 1992–2023)

*Prevalence of current use*

Prevalence of current use is calculated by dividing the number of current smokers by the total population. Prevalence of current cigarette smoking and prevalence of current smoking for combustible tobacco of cigarettes, cigars and pipe can be estimated for all waves.

*Prevalence of daily use*

Prevalence of daily use is calculated by dividing the number of daily smokers by the total population. Prevalence of daily cigarettes smoking can be calculated for all waves, while prevalence of daily smoking for combustible tobacco of cigarettes, cigars and pipe can be calculated for the 2008 wave and the 2012 to 2023 waves onwards.

*Prevalence of former use*

Prevalence of former use is calculated by dividing the number of former smokers by the total population. Prevalence of former cigarette smoking can be calculated for all waves, while prevalence of former smoking for combustible tobacco of cigarettes, cigars and pipe can be calculated from the 2008 wave onwards.

*Quit ratios*

Quit ratios indicate the proportion of ever smokers who have quit smoking. It is calculated by dividing the number of former smokers by the number of ever smokers (current and former smokers). Quit ratios, can be calculated for all waves.

*Duration of smoking*

Duration of smoking (in years) can be estimated by subtracting the initial age of regular smoking from their current age. While the duration of smoking for current smokers can be estimated for all waves since the 2012 wave, the duration of smoking for former smokers can be only estimated in the 2017 wave.

*Pack-years*

Given certain key assumptions, for instance that the tobacco consumption reported remained unchanged throughout the period the participant was a smoker, pack-years can be estimated using the information on when participants started smoking regularly and how many cigarettes participants smoked per day, which can be derived since 2012. It is calculated by dividing the number of cigarettes smoked per day by 20 (assuming one pack equals 20 cigarettes) and then multiplying by the duration of smoking, with the assumption that current smokers have always been smoking the same amount and never quit for a considerable period. Pack-years for current smokers can be estimated since the 2012 wave, although pack-years for former smokers can be only estimated in the 2017 wave.

*Time since quitting*

Time since quitting among former smokers can be computed by subtracting the age when the respondent quit smoking from the current age of the former smoker, but can be calculated only in the 2017 wave.

*Initiation rates of regular smoking*

Initiation rates can also be estimated since the 2012 wave by dividing the number of subjects who started smoking regularly during a particular period by the number of never smokers at the time.

## DISCUSSION

This study is the first systematic assessment of how tobacco and related product use has been measured in the Eurobarometer surveys on smoking/tobacco since 1992. While some inconsistencies exist in the phrasing and structure of questions and responses across waves, trends in current, daily, former, and never use of conventional cigarettes have been consistently evaluated. This consistency allows for meaningful comparisons over time.

The scope of monitoring tobacco and related product consumption has further expanded with the inclusion of e-cigarette use since the 2009 wave, various combusted products such as cigars, pipes, cigarillos, and waterpipes since the 2012 wave, and heated tobacco products (HTPs) use since the 2020 wave. However, limitations remain regarding the availability of detailed data on product-specific intensity and initiation of use. While indicators related to SHS exposure and smoking cessation have been incorporated in multiple waves, their comparability is constrained due to variations in question wording and response options.

There is broad agreement that the primary goal of tobacco control in the EU, where conventional tobacco products still dominate the market, is to achieve a continued and quick decline in prevalence of current cigarette smoking and an increase in successful cigarette quit attempts^2^. Thus, current cigarette smoking prevalence and cigarette quit ratios are key indicators of success as outlined in Articles 6 to 14 of the WHO FCTC^1^. Variations in definitions and question wording may lead to differing estimates of those indicators^17,21^.

First, definitions of current smoking vary across surveys. Eurobarometer defines current smokers based solely on whether they smoke at the time of the survey, without considering lifetime consumption or time limits. In contrast, the US National Survey on Drug Use and Health includes past 30-day use^22^, while the GATS and WHO focus on daily or occasional smoking^23^. The U.S. Centers for Disease Control and Prevention require both daily or someday use and a 100-cigarette lifetime threshold^24^. Previous studies have shown this threshold may underrepresented groups, such as women, youth and racial/ethnic minorities as social smokers, leading to underestimation of prevalence^22,25^. Additionally, the US National Health Interview Survey has suggested that the number of non-daily smokers has increased among current smokers^26^; thus highlighting the need to distinguish between current and daily smoking^27^. Other studies have also shown that the past 30-day condition may capture more light smokers compared to the question regarding daily or occasional use^28,29^. Consequently, these Eurobarometer estimates may overlook early smoking behaviors, such as experimental/social/light smoking, which were not captured by the past 30-day condition^29,30^; therefore, further research is necessary to explore factors contributing to variations in estimates based on questionnaire design.

Second, quit ratios can be computed in all waves, but have limited information on long-term success in smoking cessation. Successful cessation is a complex process, with various outcome measures used in research^31,32^. Key predictors of long-term success in cessation include age at smoking initiation, history of quit attempts, age of quitting and nicotine dependence, and the number of cigarettes smoked per day^9,32,33^; however, the comparability of these indicators across the waves is limited. Hence, incorporating that information into ongoing surveys could be useful for evaluating the success of cessation and could lead to a better understanding of the complex life-course from smoking initiation to cessation.

Achieving a continuous and rapid reduction in exposure to SHS/SHA is another key objective of tobacco regulation in the EU^2,3^. Marked changes in the wording of questions and locations related to SHS exposure were observed across the waves. SHS exposure is generally assessed by asking if respondents have seen someone smoking or been exposed to smoke^9,14^. The GATS survey adopted the former^14^, while the Eurobarometer surveys used mixed definitions, making temporal comparisons difficult and potentially introducing measurement bias in estimations^34^. For example, when few countries had smoke-free laws for bars, there was a question measuring SHS in bars. Since 2009, questions have standardized SHS exposure indoors over the past six months for more realistic estimate, whereas as compliance improved in most EU countries, recent surveys shifted to include questions on outdoor exposure. The EC tailors questions to policy relevance and survey costs, meaning SHS exposure-related questions were often shaped by the stage of policy implementation around smoke-free environments. It is important to monitor more detailed SHS exposure indicators across homes, workplaces, and indoor and outdoor public spaces, as well as distinctions between the hospitality sector and other sectors, including SHA exposure^35^.

The Eurobarometer surveys primarily focus on combustible tobacco product use, and prior to 2009, measuring the overall tobacco and nicotine use was uncertain. Since then, aggregating data on single products has allowed trends in overall tobacco and nicotine use to be assessed. These Eurobarometer surveys could be a useful tool for tracking not only single-product use but also dual and poly-use of the products. Currently, there is limited understanding of these patterns, particularly regarding the use, quantity, and frequency of major products^36^. Accurately characterizing dual and poly-use is essential to determine whether it reflects genuine product substitution affecting smoking prevalence or merely situational substitution with no real impact on smoking prevalence. Additionally, the Tobacco Products Directive includes provisions for introducing new measures as certain products, such as HTPs, become more popular. This necessitates further fine-tuning of classification integrity and capturing the trend in new tobacco products, to counter fast-changing tobacco industry strategies^37^.

Although these Eurobarometer surveys were not originally designed for tobacco control research, more than 70 peer-reviewed studies have been published, contributing to the growing evidence base on the determinants of tobacco use and policy evaluation in the EU^38,39^. Thus, using indicators from these surveys to monitor and evaluate the success of tobacco control policies could be beneficial^4^. To mitigate bias and improve data comparability, it is important to maintain consistency in measuring the same constructs, with guidance from tobacco control experts^40^. When changes in questions are needed to reflect the stage of the tobacco epidemic and policy implementation, documenting these changes along with information on how and who made them, will enhance transparency and facilitate a more precise understanding and interpretation of the survey findings for effective tobacco policy development^41^.

### Future implications

Our findings suggest several future implications. First, the key recommendation is to strike the best balance regarding indicators for monitoring based on WHO MPOWER priorities, evidence-based recommendations of tobacco control epidemiologists, and resources employed by the EC for the supplements of these Eurobarometer surveys. The survey has added value beyond the EC’s monitoring purposes; therefore, it is beneficial for various stakeholders, including policymakers and healthcare professionals. The need for data collection and monitoring reform has been previously emphasized^17,19^. These surveys are a unique source of knowledge and information in the EU due to a combination of broad topics consistently covered over time, regular publication, and geographical coverage.

### Limitations

We have used the official questionnaires and reports on the EC website dedicated to these Eurobarometer surveys. There are, however, some limitations that need to be taken into account when interpreting the findings. We have studied the official English version of the questionnaires. While official translations are available in the rest of the languages of the EU Member States, comparing the wording in other official languages was beyond the scope of this review. Hence, we could not provide the actual impact of the change in wording in other EU languages. Future comparative studies with surveys across the EU would provide a more robust explanation of the consequences of these variations.

## CONCLUSIONS

The Eurobarometer surveys provide a valuable resource for monitoring tobacco use trends across the European Union, offering insights that inform tobacco control policies. While comparability across waves is not always seamless, key indicators such as smoking prevalence and quit ratios have been consistently assessed, and the inclusion of product-specific use has expanded their scope. Periodic expert review can help maintain consistency in estimating trends in smoking patterns and timely capturing the use of emerging products, ensuring meaningful comparisons of tobacco-related indicators over time.

## Supplementary Material



## Data Availability

All data relevant to this study are based on Eurobarometer reports and questionnaires which are publicly available.

## DISCLAIMER

F.T. Filippidis, Editorial Board member of the journal, and C.I. Vardavas, Strategic Development Editor of the journal, had no involvement in the peer-review or acceptance of this article and had no access to information regarding its peer-review. Full responsibility for the editorial process for this article was delegated to a handling editor of the journal. Where authors are identified as personnel of the International Agency for Research on Cancer/World Health Organization, the authors alone are responsible for the views expressed in this article and they do not necessarily represent the decisions, policy or views of the International Agency for Research on Cancer /World Health Organization.
