# Supplementary material for: Mapping indicators of tobacco and related product use: Unveiling challenges and variations in the Eurobarometer surveys over three decades
Source: Tob Induc Dis. 2025 Apr 16;23:10.18332/tid/202651. doi: 10.18332/tid/202651 (PMC12002166; doi:10.18332/tid/202651)
Supplement: Supplementary file 1 [file TID-23-49-s1.pdf]

Supplementary

Supplementary Table 1. Wording differences of the questionnaire by tobacco and related product indicators across the Eurobarometer waves (11 waves:1992-2023), A qualitative review

Wave (Year)	Changes	Question	Responses
**Current, Former and Never use**			
1992	-	Which of the following things applies to yourself?	You smoke manufactured cigarettes<br>You smoke roll-your own cigarettes<br>You smoke cigars or a pipe<br>You used to smoke but you have stopped<br>You have never smoked
2002	Yes	Which of the following applies to you?<br><br>Do you smoke regularly, or occasionally?	You smoke packed cigarettes<br>You smoke roll-up cigarettes<br>You smoke cigars or a pipe<br>You chew tobacco or take snuff<br>You used to smoke but you have stopped<br>You have never smoked<br><br>Regularly/Occasionally
2005	No	(Same as above)	(Same as above)
2006	No	(Same as above)	(Same as above)
2008	Yes	Regarding smoking cigarettes, cigars or a pipe, which of the following applies to you?<br><br>Regarding oral tobacco such as snuff, snus or chewing tobacco, which of the following applies to you	You smoke every day<br>You smoke occasionally<br>You used to smoke but you have stopped<br>You have never smoked<br><br>You take it every day<br>You take it occasionally<br>You used to take it regularly, but you stopped<br>You have never tried it
2009	Yes	Regarding smoking cigarettes, cigars or a pipe, which of the following applies to you?	You smoke at the present time<br>You used to smoke but you have stopped<br>You have never smoked

2012	Yes	Regarding smoking cigarettes, cigars or a pipe, which of the following applies to you?	You currently smoke<br>You used to smoke but you have stopped<br>You have never smoked
2014	Yes	Regarding smoking cigarettes, cigars, cigarillos or a pipe, which of the following applies to you? In this question and the following questions in this section, smoking cigarettes does not include use of electronic cigarettes.<br><br>Regarding the use of electronic cigarettes or any similar electronic devices (e-shisha, e-pipe), which of the following statements applies to you?	You currently smoke<br>You used to smoke but you have stopped<br>You have never smoked<br><br>You currently use electronic cigarettes or similar electronic devices (e.g., e-shisha, e-pipe)<br>You used them in the past, but no longer use them<br>You tried them in the past but no longer use them<br>You have never used them
2017	No	(Same as above)	(Same as above)
2020	Yes	Regarding smoking cigarettes, cigars, cigarillos or a pipe, which of the following applies to you?<br><br>Thinking about the following products, which of the following applies to you? (E-cigarettes/ HTPs)	You currently smoke<br>You used to smoke but you have stopped<br>You have never smoked<br><br>You currently use it<br>You used to use it but you have stopped<br>You have tried only once or twice<br>You have never used it
2023	No	(Same as above)	(Same as above)

Occasional and daily use

Wave (Year)	Changes	Question	Responses
1992	-	Not collected	Not collected
2002	-	Do you smoke regularly or occasionally?	Regularly<br>Occasionally
2005	No	(Same as above)	(Same as above)
2006	No	(Same as above)	(Same as above)
2008	Yes	Regarding smoking cigarettes, cigars or a pipe, which of the following applies to you?	You smoke every day<br>You smoke occasionally<br>You used to smoke but you have stopped<br>You have never smoked

		Regarding oral tobacco such as snuff, snus or chewing tobacco, which of the following applies to you?	You take it every day<br>You take it occasionally<br>You used to take it regularly, but you stopped<br>You have tried it at least once<br>You have never tried it
2009	Yes	Do you use the following tobacco products every day, occasionally or not at all?<br>(Manufactured cigarette, Hand-rolled cigarettes, Cigars, Waterpipe (shisha, hookah)	Yes, everyday<br>Yes, occasionally<br>No, not at all
2012	Yes	How often do/did you use the following tobacco products?<br>(Boxed cigarettes, Hand-rolled cigarettes, Cigars, Pipe)	Every day<br>Weekly<br>Monthly<br>Less than monthly<br>You have tried only once or twice<br>Never<br>Refusal
2014	Yes	How often do/did you use the following tobacco products?<br>(Boxed cigarettes, Hand-rolled cigarettes, Cigars, Cigarillos, Pipe)	Every day<br>Weekly<br>Monthly<br>Less than monthly<br>You have tried only once or twice<br>Never<br>Refusal
2017	Yes	How often do you use the following tobacco products? (Boxed cigarettes, Hand-rolled cigarettes, Cigarillos, this excludes cigars, Cigars, this excludes cigarillos, Pipe)<br><br>How often did you use the following tobacco products? (Boxed cigarettes, Hand-rolled cigarettes, Cigarillos, this excludes cigars, Cigars, this excludes cigarillos, Pipe)<br><br>How often do you use electronic cigarettes or similar electronic devices (e.g. e-shisha, e-pipe)?	Every day<br>Weekly<br>Monthly<br>Less than monthly<br>You have tried only once or twice<br>Never<br>Refusal

		How often did you use electronic cigarettes or similar electronic devices (e.g. e-shisha, e-pipe)?	
2020	Yes	How often do you use the following tobacco and related products? (Boxed cigarettes, Hand-rolled cigarettes, Cigarillos, this excludes cigars, Cigars, this excludes cigarillos, Pipe, E-cigs with nicotine, E-cigs without nicotine, HTPs)<br><br>How often do you use the following tobacco and related products? (Boxed cigarettes, Hand-rolled cigarettes, Cigarillos, this excludes cigars, Cigars, this excludes cigarillos, Pipe, E-cigs with nicotine, E-cigs without nicotine, HTPs)	Every day<br>Weekly<br>Monthly<br>Less than monthly<br>You have tried only once or twice<br>Never<br>Refusal

Intensity of use

Wave (Year)	Changes	Question	Responses
1992	-	Do you smoke every day? If so, how many cigarettes a day do you smoke?	Yes, less than 5 cigarettes a day<br>5 to 9 cigarettes a day<br>10 to 14 cigarettes a day<br>15 to 19 cigarettes a day<br>20 to 24 cigarettes a day<br>25 to 29 cigarettes a day<br>30 to 34 cigarettes a day; 35 to 39 cigarettes a day<br>0 or more cigarettes a day
2002	No	(Same as above)	(Same as above)
2005	No	(Same as above)	(Same as above)
2006	No	(Same as above)	(Same as above)
2008	-	Not collected	Not collected
2009	Yes	On average, how many cigarettes do you smoke each day?<br>On average, how many cigars do you smoke each day?<br>On average, how many pipes do you smoke each day?	__ cigarettes<br><br>__ cigars<br><br>__ pipes
2012	Yes	On average, how many cigarettes do you or did you smoke each day?	__ cigarettes

2014	No	(Same as above)	(Same as above)
2017	Yes	On average, how many cigarettes do you smoke each day?<br>On average, how many cigarettes did you smoke each day?	__ cigarettes<br><br>__ cigarettes
2020	Yes	On average, how many cigarettes do you smoke each day?<br>On average, how many cigarettes did you smoke each day?<br>On average, how many heated tobacco sticks or units do you consume each day?<br>On average, how many heated tobacco sticks or units did you consume each day?	__ cigarettes<br><br>__ cigarettes<br><br>__ sticks or units<br><br>__ sticks or units
2023	Yes	On average, how many units of the following products did you smoke or use every day?<br><br>On average, how many units of the following products did you smoke or use every day?<br><br>On average, how many times do you use e-cigarettes on a daily basis?<br><br>On average, how many times did you use e-cigarettes on a daily basis?	__ units/day<br><br>__ units/day<br><br>__ number of times per day<br><br>__ number of times per day

Initiation of use

Wave (Year)	Changes	Question	Responses
1992	-	Not collected	Not collected
2002	-	Not collected	Not collected
2005	-	Not collected	Not collected
2006	-	Not collected	Not collected
2008	-	Not collected	Not collected
2009	-	Not collected	Not collected
2012	Yes	How old were you when you started smoking on a regular basis, i.e. at least once a week?	__ years old
2014	No	(Same as above)	(Same as above)

2017	No	(Same as above)	(Same as above)
2020	No	(Same as above)	(Same as above)
2023	No	(Same as above)	(Same as above)

Smoking quit attempt

Wave (Year)	Changes	Question	Responses
1992	-	Not collected	Not collected
2002	-	Not collected	Not collected
2005	-	Not collected	Not collected
2006	-	Have you tried to give up smoking in the last 12 months?<br>(IF YES) How many times have you tried to give up smoking in the last 12 months?	No never<br>Yes, between 1 and 5 times<br>Yes, between 6 and 10 times<br>Yes, more than 10 times
2008	-	Not collected	Not collected
2009	Yes	Have you tried to quit smoking in the last 12 months?	Yes, once<br>Yes, between 2 and 5 times<br>Yes, more than 5 times<br>No<br>DK
2012	Yes	Have you ever tried to quit smoking?	Yes, in the last 12 months<br>Yes, more than a year ago<br>No, never<br>DK
2014	No	(Same as above)	(Same as above)
2017	No	(Same as above)	(Same as above)
2020	Yes	Have you ever tried to quit smoking?<br>Have you ever tried to stop using e-cigs or HTPs?	Yes, in the last 12 months<br>Yes, more than a year ago<br>No, never<br>DK
2023	-	Not collected	Not collected

Duration of last quit attempt

Wave (Year)	Changes	Question	Responses

1992	-	How long is it since you gave up smoking?	Less than 5 years<br>5-9 years, 10-14 years<br>15-19 years<br>20 years or more<br>DK
2002	-	Not collected	Not collected
2005	Yes	Thinking about the last time you attempted to give up smoking, how long did this attempt last?	Less than a day<br>Between 1 and 6 days<br>Between 1 and 8 weeks<br>Over two months
2006	No	(Same as above)	(Same as above)
2008	-	Not collected	Not collected
2009	No	(Same as above)	(Same as above)
2012	-	Not collected	Not collected
2014	-	Not collected	Not collected
2017	-	Not collected	Not collected
2020	-	Not collected	Not collected
2023	-	Not collected	Not collected
**Age of quit smoking**			
Edition (Year)	Changes	Question	Responses
1992	-	Not collected	Not collected
2002	-	Not collected	Not collected
2005	-	Not collected	Not collected
2006	-	Not collected	Not collected
2008	-	Not collected	Not collected
2009	-	Not collected	Not collected
2012	-	Not collected	Not collected
2014	-	Not collected	Not collected
2017	-	How old were you when you stopped smoking?	__ years old
2020	-	Not collected	Not collected
2023	-	Not collected	Not collected
**Secondhand Smoke Exposure**			
Wave (Year)	Changes	Question	Responses

1992	-	Are there smokers or not?	At home<br>Among your friends<br>At your place of work (workshop, office, business,)<br>On journeys you make (car, bus, train, etc)<br>In places outside your home, where you go for a meal, a drink or a snack<br>In other public places to which you go regularly
		What is referred to as "passive" or "involuntary" smoking, is being exposed to other people's smoke. Whether you are a smoker or not, does this happen to you, personally?	Often<br>From time to time<br>Rarely<br>Never<br>DK
2002	-	Not collected	Not collected
2005	Yes	How long are you exposed to tobacco smoke, on a daily basis?	Never or almost never<br>Less than 1 hour a day<br>1-5 hours a day<br>More than 5 hours a day<br>DK
		How often are you bothered by exposure to tobacco in your daily life?	Very often<br>Sometimes<br>Rarely<br>Never<br>DK
2006	Yes	How long are you exposed to tobacco smoke on a daily basis?	Never or almost never<br>Less than 1 hour a day<br>1-5 hours a day<br>More than 5 hours a day<br>DK<br>(1. Indoor workplaces or offices, 2. Health care facilities, 3. Education facilities, 4. Government facilities, 5. Restaurants, pubs, or bars, 6. Theatres, cinemas or other leisure venues)
2008	Yes	Does any person living with you smoke inside your home?	You live alone<br>No one living with you smokes inside your home.<br>Someone living with you smokes inside the house

		Do you or any other person living with you smoke inside your home?	You live alone<br>You live with someone and none of you smoke inside the house<br>You live with someone and only you smoke inside the house<br>Only someone living with you smokes inside the house<br>Both you and someone living with you smoke inside the house
		At your workplace, how many hours are you exposed to tobacco smoke, on a daily basis?	More than 5 hours a day<br>1-5 hours<br>Less than 1 hour<br>Hardly ever<br>Never expose<br>I do not work outside the home
2009	Yes	How often are you exposed to tobacco smoke indoors at your workplace?	Never or almost never<br>Less than 1 hour a day<br>1-5 hour a day<br>More than 5 hours a day<br>Not relevant (don't work or don't work indoors)<br>DK
		The last time you visited in the last 6 months, were people smoking inside?	Yes<br>No<br>Have not visited in the last 6 months<br>DK<br>1. A drinking establishment such as a bar<br>2. An eating establishment such as a restaurant
2012	No	(Same as above)	(Same as above)
2014	No	(Same as above)	(Same as above)
2017	Yes	The last time you visited in the last 6 months, were people smoking inside?	Yes<br>No<br>Have not visited in the last 6 months<br>DK<br>1. A drinking establishment such as a bar<br>2. An eating establishment such as a restaurant

2020	Yes	The last time you visited in the last 6 months, were people smoking inside?	Yes<br>No<br>Have not visited in the last 6 months<br>DK<br>1. A drinking establishment such as a bar<br>2. An eating establishment such as a restaurant
		The last time you visited in the last six months, were people using e-cigs or HTPs inside?	Yes<br>No<br>Have not visited in the last 6 months<br>DK<br>1. A drinking establishment such as a bar<br>2. An eating establishment such as a restaurant<br>3. Another public space where people normally do not smoke (e.g. shopping malls, airports, concert halls)
		Still thinking about your most recent experiences in the last six months, were people smoking tobacco products?	Yes<br>No<br>Have not visited in the last 6 months<br>DK<br>1. On an outdoor terrace of a drinking or eating<br>2. At outdoor events (e.g. open-air concerts, sporting events)<br>3. In outdoor spaces intended for use by children or adolescents (e.g. nursery and school courtyard, playgrounds)
2023	Yes	In (OUR COUNTRY) and in the last six months, were people smoking in …. ? (this question refers to smoking tobacco products such as cigars, cigarettes, cigarillos, pipes but not using heated tobacco products.)	Yes<br>No<br>Have not visited in the last 6 months<br>DK<br>1. Indoor public spaces where people normally do not smoke (e.g. restaurants, bars, shopping malls, airports, concert halls)<br>2. An outdoor terrace of a drinking or eating establishment<br>3. Outdoor spaces intended for use by children or adolescents (e.g. nursery and school courtyard, playgrounds)<br>4. Public spaces (e.g., park, beach, entrance to public buildings)

		Have you ever been exposed to e-cigarettes?	5. Open air public transportation stations (e.g. bus, tram or train stations)
		Have you ever been exposed to heated tobacco products (HTPs) or nicotine pouches?	Yes, I have been offered e-cigarettes by colleagues, friends or family<br>Yes, I have seen them as part of a promotion or advertisements<br>Yes, I know many people who use e-cigarettes<br>No, I am not familiar with this type of product<br>Don't know<br><br>Yes, I have been offered heated tobacco products or nicotine pouches by colleagues, friends or family<br>Yes, I have seen them as part of a promotion or commercial<br>Yes, I know many people who use heated tobacco products or nicotine pouches<br>No, I am not familiar with these products<br>Don't know
**Health warning labels**			
Wave (Year)	Changes	Question	Responses
1992	-	Not collected	Not collected
2002	-	Do you think that the health warnings printed on the packets are effective in persuading people to smoke less or to give up smoking? (some examples of warnings are "smokers die younger" or "smoking causes fatal lung cancer")	Is harmless/ can cause discomfort<br>can cause some health problems such a as respiratory problems<br>can even, in the long term, cause serious illnesses such as cancer<br>it depends<br>Don't know
2005	-	Not collected	Not collected
2006	Yes	Thinking about the health messages that are on tobacco packs, have these messages been very effective, somewhat effective, not very effective or not effective at all?<br><br>1. Informing you about the health effects of tobacco<br>2. Persuading you not to start smoking again	Very effective<br>Somewhat effective<br>Not very effective<br>Not at all effective<br>Don't know
2008	-	(Same as above)	(Same as above)
2009	-	Not collected	Not collected

2012	Yes	Thinking about the health warnings that are used on tobacco packs in our country, would you say that these warnings have/have had an impact on your attitude and behaviour towards smoking?	Yes, they encouraged you to quit<br>Yes, they encouraged you to smoke less<br>Yes, they increased your awareness of the health effects of tobacco, and you tried to change your behaviour but did not succeed<br>Yes, they increased your awareness of the health effects of tobacco, but did not convince you to change your behaviour<br>No, they did not have any impact on you
		Do you think that the health warnings used on tobacco packs in our country help young people not to start smoking?	Yes, definitely<br>Yes, probably<br>No, probably not<br>No, definitely not
2014	-	Not collected	Not collected
2017	-	Not collected	Not collected
2020	-	Not collected	Not collected
2023	-	Not collected	Not collected

Footnote: The column indicating whether there has been a change or not is marked “Yes” if there has been a change in either the question or the answer compared to the previous wave and “No” if there has been no change. If there is no change, the question-and-answer columns are left blank to avoid repetition.

Supplementary Table 2. Wording differences of the questionnaire of sociodemographic and socio-economic status information across the Eurobarometer waves (11 waves:1992-2023), A qualitative review

Questionnaire										
**Sex**	**1992**	**2002**	**2005**	**2006**	**2008**	**2009**	**2012**	**2014**	**2017**	**2020**
Q. Gender		■	■	■	■	■	■	■	■	■
A. Male or Female		■	■	■	■	■	■	■	■	■
* None of the above/ Non binary/ do not recognize yourself in above categories were introduced in 2020										
**Age**	**1992**	**2002**	**2005**	**2006**	**2008**	**2009**	**2012**	**2014**	**2017**	**2020**
Q. How old are you?		■	■	■	■	■	■	■	■	■
A. ___ years old		■	■	■	■	■	■	■	■	■
**Area of residence**	**1992**	**2002**	**2005**	**2006**	**2008**	**2009**	**2012**	**2014**	**2017**	**2020**
Q. Would you say you live in a…?		■	■	■	■	■	■	■	■	■
A. Rural area or village/ Small or middle-sized town/Large town/ DK		■	■	■	■	■	■	■	■	■
**Marital Status**	**1992**	**2002**	**2005**	**2006**	**2008**	**2009**	**2012**	**2014**	**2017**	**2020**
Q. Could you give me the letter which corresponds best to your own current situation?		■	■	■		■	■			
Q. Which of the following best corresponds to your own current situation?								■	■	■
A. Married/ Remarried/ Unmarried, currently living with partner/ Unmarried, having never lived with a partner/ Unmarried, having previously lived with a partner, but now on my own/ Divorced/ Separated/ Widowed/ Other/Refusal		■	■	■						
A1. Married or remarried (Living without children/ Living with the children of a previous marriage/ Living with the children of this marriage and of a previous marriage)						■	■	■	■	■

A2. Single living with a partner (Living without children/ Living with the children of this union/ Living with the children of this marriage and of a previous union)<br>A3. Single (Living without children/ Living with children)<br>A4. Divorced or separated (Living without children/ Living with children)<br>A5. Widow (Living without children/ Living with children)<br>A6. Other/ Refusal										
**Occupation**	**1992**	**2002**	**2005**	**2006**	**2008**	**2009**	**2012**	**2014**	**2017**	**2020**
Q. What is your current occupation?		■		■		■	■	■	■	■
A. Responsible for ordinary shopping and looking after the home, or without any current occupation, not working/ Student/ Unemployed or temporarily not working/ Retired or unable to work through illness/ Farmer/ Fisherman/ Professional (lawyer, medical practitioner, accountant, architect)/ Owner of shop, craftsmen, other self-employed person/ Business proprietors, owner (full or partner) of a company/ Employed professional (employed doctor, lawyer, accountant, architect)/ General management, director or top management (managing directors, director general, other director)/ Middle management, other management (department head, junior manager, teacher, technician)/ Employed position, working mainly at a desk/ Employed position, not at a desk but travelling (salesmen, driver, etc.)/ Employed position, not at a desk, but in a service job (hospital, restaurant, police, fireman)/ Supervisor/ Skilled manual worker/ Other (unskilled) manual worker, servant		■		■		■	■	■	■	■
Q. As far as your current occupation is concerned, would you say you are self-employed, an employed, a manual worker or would you say that you are without a professional activity? Does it mean that you are a …					■					
**Education**	**1992**	**2002**	**2005**	**2006**	**2008**	**2009**	**2012**	**2014**	**2017**	**2020**
Q. How old were you when you stopped full-time education?		■	■	■	■	■	■	■	■	■
A. ___years old/ Still in full time education		■	■	■						
A. ___years old/ Still in full time education/ Never been in full time education/ Refusal or no answer					■	■	■	■	■	■
**Difficulties to paying bills**	**1992**	**2002**	**2005**	**2006**	**2008**	**2009**	**2012**	**2014**	**2017**	**2020**

Q. During the last twelve months, would you say you had difficulties to pay your bills at the end of the month?	■	■	■	■	■
A. Most of the time/From time to time/Almost never or never/ Refusal	■	■	■	■	■

© 2025 Teshima A. et al.
